# Hypoxia, Hypoxia-Inducible Factor-1α, and Innate Antileishmanial Immune Responses

**DOI:** 10.3389/fimmu.2018.00216

**Published:** 2018-02-22

**Authors:** Valentin Schatz, Patrick Neubert, Franz Rieger, Jonathan Jantsch

**Affiliations:** ^1^Institute of Clinical Microbiology and Hygiene, University Hospital of Regensburg, University of Regensburg, Regensburg, Germany

**Keywords:** hypoxia-inducible factor 1, leishmaniasis, macrophages, oxygen, *Nos2*, hypoxia

## Abstract

Low oxygen environments and accumulation of hypoxia-inducible factors (HIFs) are features of infected and inflamed tissues. Here, we summarize our current knowledge on oxygen levels found in *Leishmania*-infected tissues and discuss which mechanisms potentially contribute to local tissue oxygenation in leishmanial lesions. Moreover, we review the role of hypoxia and HIF-1 on innate antileishmanial immune responses.

## Introduction

Low oxygen (O_2_) environments are a key feature of infected and inflamed tissue. Several lines of evidence demonstrate that oxygen levels of afflicted tissues are much lower than these currently used in standard cell culture experiments and, in general, correspond to values below 4% O_2_ [reviewed in Ref. (1, 2)] Low oxygen levels are able to incapacitate oxygen-dependent antimicrobial effector enzymes such as the phagocytes oxidase or inducible NO synthase which both require oxygen as cosubstrate in order to produce their antimicrobial reactive oxygen species (ROS) and reactive nitrogen species (RNS) [reviewed in Ref. (1, 2)]. Moreover, hypoxia is not only a state of reduced availability of oxygen but also in addition induces a transcriptional response, which is governed by the transcription factors (TFs) hypoxia-inducible factor (HIF)-1 and HIF-2. Both TFs belong to the basic helix–loop–helix-PAS family of TF, consisting of HIF-1α or HIF-2α and its dimerization partner aryl hydrocarbon receptor nuclear translocator (ARNT) [reviewed in Ref. (3)]. Prolyl-hydroxylase domain (PHD) enzymes play a key role in the regulation of HIF-1α and HIF-2α since oxygen is a critical substrate for the PHD enzymes. Under conditions of ample oxygen, they hydroxylate HIF-α subunits that target HIF-α in a von Hippel-Lindau tumor suppressor-dependent manner to proteasomal degradation [reviewed in Ref. (4–6)].

Subsequent studies revealed that HIF-1α is not only involved in adaption of cells to low oxygen environments but that this TF is also stabilized upon infectious and inflammatory stimuli under conditions of ample oxygen as well. Furthermore, HIF-1α is required for inflammatory responses of innate immune cells *in vitro* and *in vivo* [reviewed in Ref. (1, 7–11)]. Mechanistically, normoxic, inflammatory HIF-1α stabilization is closely linked to nuclear factor (NF)-κB activation (12, 13), and involves transcriptional and posttranslational signaling events (14–16). Altogether, these findings demonstrate that hypoxic and inflammatory responses are intertwined.

Therefore, there is a growing interest to uncover the impact of hypoxia and HIF-1α in infectious diseases and its impact on host–pathogen interaction [reviewed in Ref. (1, 2, 17–21)]. In this review, we will summarize the evidence of hypoxia and the TF HIF-1α and its impact on innate immune responses directed against infection with *Leishmania major, Leishmania amazonensis*, and *Leishmania donovani*, which are able to cause cutaneous, mucocutaneous and systemic (visceral) diseases, respectively (Table 1).

**Table 1:** Role HIF-1α in mononuclear phagocytes in Leishmaniasis.

*Leishmania* species	Type of disease	Tissue tropism	Role of HIF-1α in mononuclear phagocytes	
			*In vitro*	*In vivo*
*L. major*	Cutaneous leishmaniasis	(Local) skin	Parasite control	Induction of *Nos2*
				Cutaneous control of parasites

*L. amazonensis*	Mucocutaneous leishmaniasis	Skin with diffuse chronic progression	Parasite survival	Unknown

*L. donovani*	Visceral leishmaniasis	Spleen, bone marrow, liver	Parasite survival	Induction of MDSC
				Propagation of parasites

## Oxygen Level in Leishmanial Lesions

In guinea-pigs, intravenous challenge experiments with guinea-pig adapted *Leishmania enrietti* demonstrate that skin tissue predisposes to the development of leishmanial lesions (22). Given that skin tissue is known to display low oxygen levels (23–25), these data suggest that low oxygen microenvironments might provide a safe haven for *Leishmania* parasites (22). Araújo et al. assessed lesional tissue oxygen levels with an immunohistochemical method in cutaneous *L. amazonensis* infection (26). For that purpose, they injected a 2-nitroimidazol derivative into *L. amazonensis* infected mice. Upon injection, these compounds are enriched in tissues with very low oxygen tensions and form adducts that can be visualized after staining with adduct-specific antibodies (27, 28). Using this technology, they found that in *L. amazonensis-*induced lesions very low oxygen tensions are present (26).

To the best of our knowledge, there are no data available on tissue oxygen levels after infection with *L. donovani* in liver tissue. Since it is known that steep oxygen gradients exist in the liver (27), it is very likely that *L. donovani*-infected liver tissues display low oxygen levels as well. Using 2-nitroimidazol based techniques to visualize hypoxic tissues, Hammami et al. revealed that in *L. donovani*-infected spleens, very low tissue oxygen prevail (29).

Although staining of hypoxic areas with 2-nitroimidazol-derivatives allows for identification of severely hypoxic tissue, this method does not provide quantitative data and is not suitable for continuous recording of lesional tissue oxygen levels (25). Alternative methodologies to quantitatively assess tissue oxygen levels rely on polarographic oxygen sensors or imaging of tissue oxygenation by using oxygen-dependent luminescence quenching (25). For instance, non-invasive luminescence-based oxygen imaging using sensor foils can be used to quantitatively assess tissue oxygenation in a non-invasive manner over time (25). Using this technology in a mouse model of self-healing cutaneous leishmaniasis, Mahnke et al. observed that oxygen tension in leishmanial skin lesions displayed oxygen levels around ~2.8% O_2_ when the lesions reached their maximum size (30). Resolution and healing of the lesion was paralleled by an increase of tissue oxygenation (30). Altogether, these findings indicate that low oxygen levels prevail in *Leishmania*-infected tissue.

### Potential Factors Regulating Tissue Oxygen Levels in *Leishmania*-Infected Tissues

**Figure 1:**
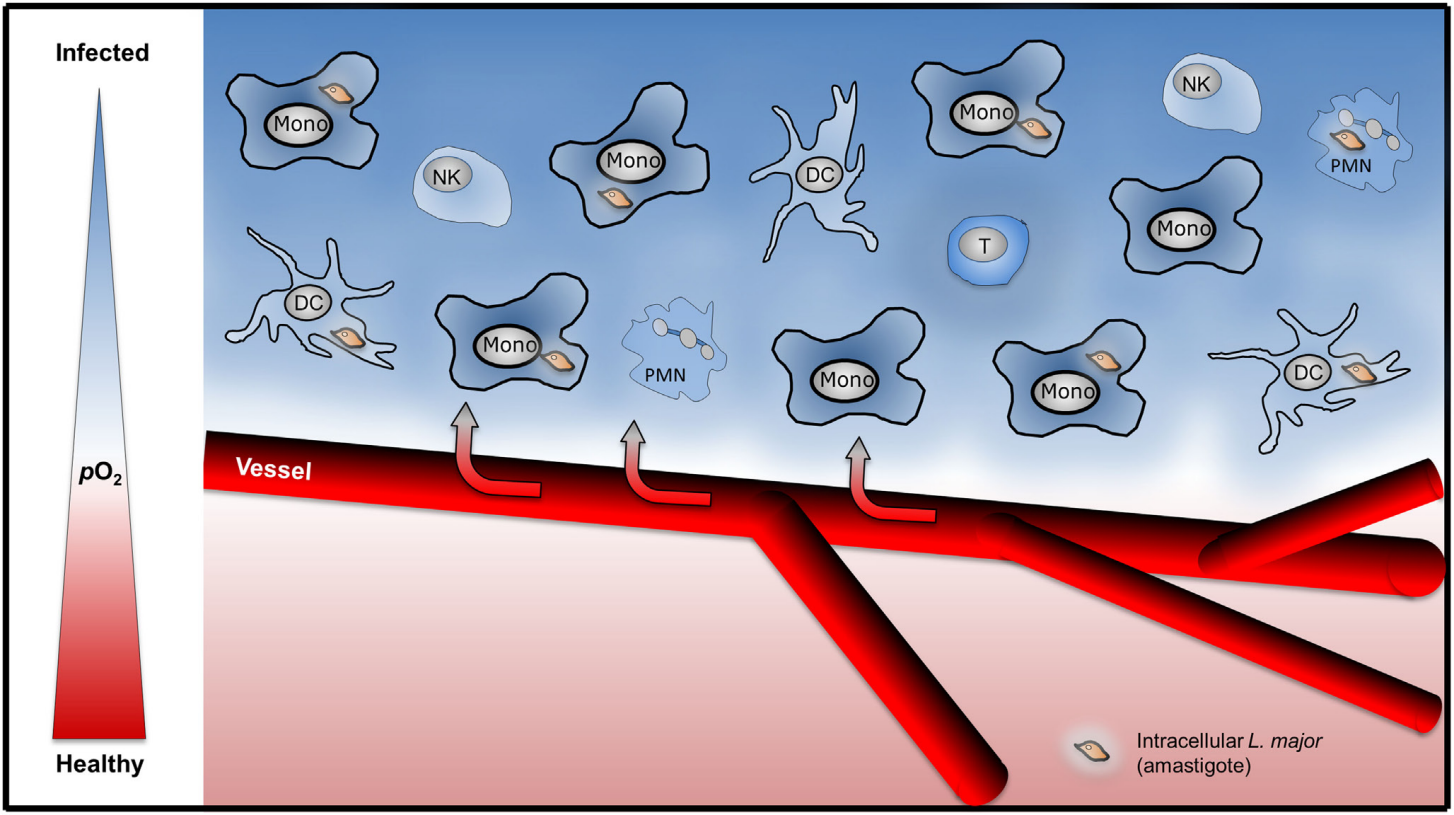
Infiltration of immune cells might drive low tissue oxygenation in *Leishmania major*-infected skin. Upon *L. major*-infection, infiltration of granulocytes [polymorphonuclear granulocyte (PMN)], monocytes (Mono), dendritic cells (DC), NK cells, and T cells might induce increased local consumption of O_2_ resulting in hypoxic tissue O_2_ levels.

The mechanisms that drive low oxygen levels in *Leishmania* infected tissue are, however, largely unknown. Recent studies demonstrate that influx of granulocytes results in increased O_2_ demand which ultimately results in low tissue oxygen levels (31). Low tissue oxygenation in *L. major*-infected tissue might result from either (i) infection-induced disturbance of local tissue perfusion or (ii) an increased local O_2_ consumption by the infectious agent and/or infiltrating immune cells (Figure 1). However, the contribution of infiltrating granulocytes, monocytes, NK cells, and T cells on tissue oxygen levels in *Leishmania* infected tissues is unexplored. Moreover, *Leishmania* might interfere with inflammation induced angiogenesis. For instance, *Leishmania* is able to scavenge angiogenic factors such as VEGF-A. Thereby *Leishmania* might interfere with inflammation-associated angiogenesis and disturb local oxygen supply (32). Nevertheless infection with *L. major* eventually triggers a vascular endothelial growth factor (VEGF)-A/VEGF receptor (VEGFR)-2-dependent proliferation of endothelial cells (33). Underscoring the potential important role of VEGF-A/VEGFR-2 signaling, Araújo and Giorgio suggest that VEGF-A levels upon infection might predict the outcome of *L. amazonensis-*infection. They recorded higher VEGF-A levels in healer mice compared to non-healer mice (34). Since VEGF-A is a known HIF-1α target (35) and HIF-1α is present in *L. amazonensis-* and *L. major*-infected lesions in humans and in preclinical models (26, 33, 34, 36), it is possible that HIF-1α plays a role in infection-induced proliferation of the endothelial cells. However, to the best of our knowledge, this has not been tested in *L. major* and *L. amazonensis* mouse models yet. In addition to maintaining oxygenation of infected tissue, endothelial cell proliferation might curtail *L. major* induced-inflammatory responses (37). The activity of endothelial NO synthase plays an important role in endothelial cell mediated anti-inflammatory activity (38–40). Endothelial cell mediated anti-inflammatory activity might reduce the influx of immune cells and/or their O_2_-consumption, thereby increase tissue oxygen levels and facilitate subsequent resolution of disease. Since endothelial NO synthase expression is linked to HIF-1α-accumulation (41), endothelial HIF-1α might promote anti-inflammatory properties in endothelial cells as well. Further studies, however, are required to investigate this issue. Earlier findings demonstrate that the “myeloid cell differentiation antigen carcinoembryonic antigen-related cell adhesion molecule 1” (CEACAM1) is involved in angiogenesis and antileishmanial control (42). Whether CEACAM1-dependent signaling affects VEGF-A/VEGFR and/or HIF-1α signaling in this state of affair is unknown. Further investigations are required to understand the interplay of inflammation-triggered angiogenesis, enhanced O_2_ demand in inflamed tissues, and its impact on overall tissue oxygenation in leishmaniasis.

## Impact of Hypoxia on Host–Pathogen Interaction

### Internalization of *Leishmania* and Hypoxia

After transmission of *Leishmania* by its vector, myeloid cells are the main host cell [reviewed in Ref. (43–45)]. Hypoxic conditions are known to modulate the phagocytic/endocytic uptake of mononuclear phagocytes (46, 47). However, to the best of our knowledge, the impact of oxygen levels below 4% O_2_ on *Leishmania* uptake or internalization is unexplored.

### Polymorphonuclear Granulocytes (PMN), Hypoxia, and *Leishmania*

Polymorphonuclear granulocytes are quickly attracted to the site of infection. Dependent on the *Leishmania* species and the host’s genetic background, they play a rather detrimental role in cutaneous leishmaniasis. PMN are inefficient in killing promastigotes and undergo apoptosis which hampers the proper activation of recruited dendritic cell (DC) [reviewed in Ref. (44, 48)]. In general, PMN are relatively short-lived cells, whose survival, however, is prolonged in NF-κB- and HIF-1α-dependent manner under hypoxic conditions. Moreover, hypoxia induces the production of macrophage inflammatory protein-1β which further facilitates neutrophil survival (49). In line with this, Monceaux et al. demonstrated recently that anoxic conditions prolong PMN survival (50). Therefore, hypoxia most likely favors influx and lifespan of PMN in leishmaniasis as well. Since these cells are not able to eliminate *Leishmania*, this might ultimately prolong and/or exacerbate the disease. However, to the best of our knowledge, there is no detailed analysis on the specific role of hypoxia on PMN–*Leishmania* interaction.

### DC, Hypoxia, and *Leishmania*

Upon *Leishmania* exposure, DC phagocytose the parasite, become activated and establish an antileishmanial T cell response and thereby critically contribute to the resolution of disease [reviewed in Ref. (51)]. For instance, priming and induction of *Leishmania*-specific T_H_1 response results in secretion of IFN-γ which is required for antileishmanial immunity [reviewed in Ref. (44)]. In addition, even after resolution of disease, DC still contain viable parasites which are able to maintain antigen-specific T cell responses against *L. major*. This contributes to the long term protection against the parasites [reviewed in Ref. (51)]. Although, there is evidence that oxygen levels impact on DC functions, there are to the best of our knowledge only limited reports on the contribution of hypoxia on DC-mediated establishment of antileishmanial T cell immunity. Hypoxia might impact on DC viability (52), migratory capacity (53), maturation, and antigen presentation (54–56).

### NK Cells, Hypoxia, and *Leishmania*

In early phase of *Leishmania* infection, NK cells are activated by IL-12 and IL-18. These activated NK cells contribute to parasite control through production of IFN-γ [reviewed in Ref. (57)]. NK cells respond to hypoxia by downregulation of surface markers NKp46, NKp30, NKp44, NKG2D, and CD16, decreased cytotoxic and antiviral activity, and reduced IFN-γ expression (58–60). In contrast to these findings, Krzywinska et al. observed no mitigated IFN-γ secretion by NK cells under hypoxia (61). However, the impact of hypoxia on NK-mediated antileishmanial defense is unexplored and requires further studies.

### Monocytes/Macrophages, Hypoxia, and *Leishmania*

A few days after *L. major* and *L. donovani* infection, recruited monocytes are highly abundant in afflicted tissues (62, 63). Inflammatory monocytes are able to upregulate their ROS production in the early stage of infection with *L. major* and *L. donovani* (64, 65). However, in inflammatory monocytes infected with *L. major*, ROS production does eliminate the parasites (63). Moreover, *L. donovani*, for instance, has the ability to overcome the ROS-mediated apoptosis of host macrophages *via* induction of suppressor of cytokine signaling proteins (66). It is established that IFN-γ and TNF are two key cytokines that empower macrophages (67–69) and monocytes (63) to clear *Leishmania*. In the mouse system this results in a robust induction of the antileishmanial effector enzyme, the inducible or type 2 NO synthase (iNOS or NOS2). A high level of NO production is prerequisite of the cutaneous defense against *Leishmania major* [reviewed in Ref. (70, 71)]. Moreover, in mouse models of visceral leishmaniasis, *Nos2* expression is important for control of *L. donovani* in the liver (72), and contributes to the containment of *L. donovani* (73) in the spleen.

For induction of NOS2 and production of high level of leishmanicidal NO, costimulation of macrophages with TNF plus IFN-γ is required (63, 68, 72, 74–78). Low oxygen conditions, however, impair NO production of activated macrophages because the inducible NO synthase activity critically hinges on the availability of O_2_ as a substrate (79–81). Accordingly, under oxygen conditions below 4% O_2_ NO production of activated macrophages is diminished (30). *L. major* possesses a globin-coupled heme containing oxygen sensor soluble adenylate cyclase which allows for its adaption to hypoxic conditions (82). Hence, while *L. major* is able to adjust to hypoxia, low oxygen conditions impair the NO-dependent antileishmanial potential of macrophages resulting in impaired clearance and survival of *L. major* in activated macrophages under low oxygen conditions. However, leishmanicidal activity is restored when the cells are reoxygenated (30). These data demonstrate that local tissue oxygen levels—at least transiently—do not match the macrophages’ O_2_ demand to fight against *L. major*.

In visceral leishmaniasis, parasites express high tissue tropism toward monocytes in the spleen and bone marrow. *L. donovani*, e.g., induces expansion of hematopoietic stem cell-like mononuclear cells from bone marrow which serve as host cells for the parasite (62). Compared to the role of *Nos2* in the defense against visceral leishmaniasis in the liver (72), the contribution of *Nos2* in the control of parasite load in the spleen in visceral leishmaniasis is less pronounced. While there was no induction of *Nos2* in spleen macrophages upon infection of hamsters with *L. donovani* (83), *Nos2* expression, nevertheless, contributed to the parasite-containment in the spleen of *L. donovani* and *L. infantum-*infected mice (73, 84). In preclinical models of visceral leishmaniasis, the impact of local oxygen levels on *Nos2*-mediated antileishmanial defense and NO production in the spleen and liver is, however, to the best of our knowledge unexplored. It is tempting to speculate that low oxygen levels found in *L. donovani*-infected spleen tissue (29) incapacitate the *Nos2*-dependent antileishmanial defense in the spleen and, thus provide a safe niche for persistence and proliferation of *L. donovani*.

In contrast to *L. major*, exposure of *L. amazonensis-*infected macrophages to O_2_ levels of 5% and 1% O_2_ resulted in enhanced clearance of intracellular parasites (85, 86). These findings suggest that either (1) NO-independent mechanisms are involved in control of *L. amazonensis* under hypoxic conditions and/or (2) *L. amazonensis* is not able to adjust to low oxygen conditions. For instance, the divergent effect of hypoxia on the survival of members of the genus *Leishmania* in mononuclear phagocytes under hypoxia might be due to different metabolic requirements and O_2_-demands of the respective *Leishmania* species investigated. While it is known that *L. major* and *L. donovani* are able to adjust to hypoxic conditions by increasing their rate of glycolysis (87, 88), to the best of our knowledge detailed investigations on the intermediary metabolism of *L. amazonensis* upon exposure to low oxygen conditions are lacking.

## Mononuclear Phagocytes, HIF-1α, and Antileishmanial Immunity

In addition to low oxygen conditions, various pathogens and pathogen-derived molecules are able to induce HIF-1α even in the presence of ample oxygen [reviewed in Ref. (1, 8–17)]. Inflammatory HIF-1α accumulation under normoxic conditions critically hinges on NF-κB activation (12, 13). HIF-1α transactivation potential, however, is highly contextual. For instance, hypoxic and inflammatory HIF-1α activation, both, result in activation of glycolytic HIF-1α-target genes while only inflammatory HIF-1α activation results in robust induction of inflammatory target genes such as *Nos2* (89).

### *Leishmania* and Stabilization of HIF-1α in the Presence of Ample Oxygen

Currently, there is no consensus on whether *Leishmania* on its own is able to induce HIF-1α accumulation under normoxic conditions (Table 1). Infection of mouse macrophages *in vitro* with *L. amazonensis* and *L. donovani* triggers HIF-1α accumulation under normoxic conditions without requiring any additional inflammatory signals (90, 91). Blockade of this *Leishmania*-induced HIF-1α accumulation with cadmium chloride or RNAi results in impaired *Leishmania* recovery under normoxic conditions (90–93). In contrast to these findings and in contrast to stimulation with *Toll*-like receptor ligands [reviewed in Ref. (1, 8, 9)] or infection with various pathogens such as *S. pyogenes* (35, 94), *M. tuberculosis* (95), *Histoplasma capsulatum* (96), infection of mouse macrophages with *L. major* does not induce HIF-1α accumulation on its own but requires exogenous stimulation by IFN-γ and/or LPS (36). In line with this, Hammami et al. demonstrated that upon *L. donovani*-infection HIF-1α accumulation in CD11c^+^ mouse mononuclear phagocytes *in vivo* requires interferon regulatory factor-5-dependent inflammatory signaling (97). This suggests that *L. donovani* infection on its own is not sufficient to induce HIF-1α accumulation in mouse mononuclear phagocytes *in vivo* as well (97).

The different potential of various *Leishmania* species to induce HIF-1α accumulation in mononuclear phagocytes is unclear. HIF-1α accumulation in mononuclear cells might, for instance, help *Leishmania* to fuel its metabolic needs (98, 99). Mechanistically, the differential ability of certain *Leishmania* species to interfere with NF-κB activation (100–102) might underlie the divergent ability of different *Leishmania* species to block infection-associated inflammatory HIF-1α-accumulation under normoxic conditions. However, further studies are required to assess this issue.

### Role of HIF-1α Expression in Mononuclear Phagocytes

Earlier findings demonstrate a requirement of HIF-1α for NOS2-induction (89, 94, 103–105). In line with this, LPS/INFγ-coactivated HIF-1α-deficient macrophages displayed a diminished NOS2-dependent production of NO under normoxic conditions and impaired killing of *L. major in vitro*. Conditional targeting of HIF-1α in macrophages revealed that HIF-1α-activation is required for antileishmanial defense against cutaneous *L. major*-infection under normoxic conditions (36). This was paralleled by a blunted induction of NOS2 in lesional myeloid cells (36). However, it remains unclear whether in addition to *Nos2* other HIF-1α-regulated targets are involved in cutaneous containment of *Leishmania* as well.

While HIF-1α in mononuclear phagocytes is protective in *L. major*-induced cutaneous leishmaniasis, HIF-1α expression in these cells emerged as detrimental factor in visceral leishmaniasis. Hammami et al. used CD11c Cre-deleter mice to target HIF-1α in Cd11c^+^ mononuclear phagocytes and to test the contribution of HIF-1α in a *L. donovani*-induced preclinical model of visceral leishmaniasis (29). In line with an inhibitory role of HIF-1α in DC-mediated T cell responses (106), Hammami et al. provide evidence that in *L. donovani-*infected mice HIF-1α in CD11c^+^ cells limits the expansion of short lived effector CD8^+^ T cells and thereby exacerbates disease at early time points after infections (29). In a recently published follow-up study, they demonstrate that HIF-1α in CD11c^+^ cells limits the frequency and numbers of granulocyte–monocyte progenitors and inflammatory monocytes. Moreover, HIF-1α in CD11c^+^ cells enhances the inhibitory function of splenic mononuclear phagocytes while this TF is not involved in tissue neovascularization and splenomegaly (29). Of note, Hammami et al. demonstrate that HIF-1α negatively impacts on the expression of *Nos2* in *L. donovani*-infected mononuclear phagocytes. Discrepancies of this finding to the published literature on the requirement of HIF-1α on *Nos2*-expression and NO production might be caused by the different context of HIF-1α stabilization (29). This issue, however, demands further detailed investigation. Overall, the data suggest that HIF-1α in CD11c^+^ cells favors a myeloid-derived suppressor cell-like (MDSC) behavior of these cells. These findings were paralleled by an impaired antileishmanial control in the bone marrow of *L. donovani*-infected mice at late time points (29).

Taken together, these findings demonstrate that HIF-1α expression in CD11c^+^ mononuclear phagocytes favors development of visceral leishmaniasis in spleen and bone marrow, while HIF-1α in LysM^+^ myeloid cells supports defense against cutaneous *L. major* infection (Table 1). These findings underscore that the role of HIF-1α in myeloid cells is highly dependent on the respective context. HIF-1α is known to promote proinflammatory function of mononuclear phagocytes (35, 36, 94, 95, 105). However, the very same TF is able to induce regulatory and immunosuppressive functions in mononuclear phagocytes as well (107–109). Therefore, it is very likely that the differential context of HIF-1α stabilization in visceral and cutaneous leishmaniasis impacts on the functional consequence of HIF-1α activation. Further studies are needed to uncover the distinct signals that ultimately result in differential HIF-1α-dependent innate immune cell function.

## Author Contributions

VS, PN, and FR: contributed to writing of the manuscript. PN: provided the figure. JJ: conception of the manuscript, together with VS, PN, and FR writing of the article.

## Conflict of Interest Statement

The authors declare that the research was conducted in the absence of any commercial or financial relationships that could be construed as a potential conflict of interest.
